# Did Amino Acid Side Chain Reactivity Dictate the Composition and Timing of Aminoacyl-tRNA Synthetase Evolution?

**DOI:** 10.3390/genes12030409

**Published:** 2021-03-12

**Authors:** Tamara L. Hendrickson, Whitney N. Wood, Udumbara M. Rathnayake

**Affiliations:** Department of Chemistry, Wayne State University, 5101 Cass Avenue, Detroit, MI 48202, USA; whwood@chapman.edu (W.N.W.); rathnayake.udumbara@gmail.com (U.M.R.)

**Keywords:** aminoacyl-tRNA synthetase, evolution

## Abstract

The twenty amino acids in the standard genetic code were fixed prior to the last universal common ancestor (LUCA). Factors that guided this selection included establishment of pathways for their metabolic synthesis and the concomitant fixation of substrate specificities in the emerging aminoacyl-tRNA synthetases (aaRSs). In this conceptual paper, we propose that the chemical reactivity of some amino acid side chains (e.g., lysine, cysteine, homocysteine, ornithine, homoserine, and selenocysteine) delayed or prohibited the emergence of the corresponding aaRSs and helped define the amino acids in the standard genetic code. We also consider the possibility that amino acid chemistry delayed the emergence of the glutaminyl- and asparaginyl-tRNA synthetases, neither of which are ubiquitous in extant organisms. We argue that fundamental chemical principles played critical roles in fixation of some aspects of the genetic code pre- and post-LUCA.

## 1. Introduction

The Central Dogma, including machinery for mRNA-guided, ribosomal protein translation, had already been defined at the time of the last universal common ancestor (LUCA) [1,2]. Selection of the 20 commonly encoded amino acids had occurred as well, with selenocysteine and pyrrolysine being added to the protein code post-LUCA [3,4,5,6,7]. Consequently, at the time of LUCA, mechanisms existed to produce at least one aminoacylated tRNA per commonly coded amino acid, with these aa-tRNAs connecting the codons in the genetic code to the amino acids in proteins [8]. Nevertheless, LUCA putatively contained only eighteen specific, canonical aaRSs, divided into two classes (class I and class II) based on the structures of their active sites. These enzymes were each capable of specifically attaching their cognate amino acid to the corresponding cognate isoacceptor set of tRNAs. This set of canonical aaRSs included two copies of lysyl-tRNA synthetase, one in each class, a notable example of convergent evolution [9,10]. LUCA also contained two non-discriminating (ND) aaRSs: ND-aspartyl- and ND-glutamyl-tRNA synthetase [8]. These enzymes not only aminoacylated their cognate tRNAs, tRNA^Asp^ and tRNA^Glu^, they also misacylated tRNA^Asn^ and tRNA^Gln^ to produce Asp-tRNA^Asn^ and Glu-tRNA^Gln^. These misacylated tRNAs were converted to their cognate aminoacyl-tRNAs through the function of an amidotransferase, ADT [11].

**Figure 1:**
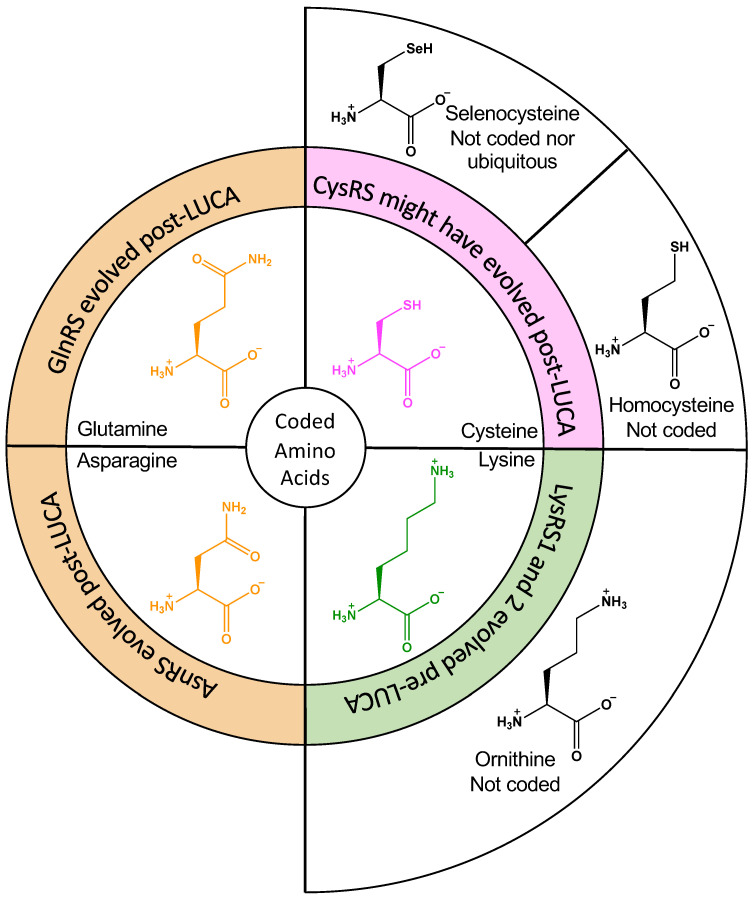
Connections between aaRS evolutionary events and amino acid substrates. Amino acid chemistry might explain idiosyncrasies in the evolution of AsnRS, GlnRS, CysRS, and LysRS. The central ring shows the amino acid substrates for these aaRSs. LysRS existed in two evolutionarily distinct forms (LysRS1 and LysRS2, green) in LUCA; CysRS is missing from some methanogenic archaea and might have been fixated post-LUCA (magenta); GlnRS and AsnRS clearly emerged post-LUCA and are still missing in many microorganisms. The outer circle shows structurally similar amino acids that were available metabolically but were rejected from the genetic code (ornithine and homocysteine) or entered the code post-LUCA (selenocysteine).

In this article, we will focus on the emergence of six aaRSs—all of which were either absent, non-discriminating, or duplicated in the most likely LUCA—namely the glutamyl- aspartyl-, glutaminyl-, asparaginyl-, cysteinyl-, and lysyl-tRNA synthetases (ND-GluRS, ND-AspRS, GlnRS, AsnRS, CysRS, and LysRS). We will briefly summarize what is known and hypothesized about the early evolution of each of these enzymes and propose scenarios wherein amino acid side chain reactivity and/or the metabolic availability of similar amino acids might have contributed to divergent evolutionary patterns. Figure 1 focuses on the four aaRSs that were either duplicated or missing in LUCA (LysRS, CysRS, AsnRS, and GlnRS). Of these, only LysRS definitively evolved pre-LUCA; however, it emerged in two forms, the expected class II enzyme and as a class I enzyme as well [9,10]. GluRS and AspRS have been omitted from this figure because non-discriminating variants of these enzymes existed at the time of LUCA and were specifically tied to the delayed emergence of GlnRS and AsnRS [11]. In each case, we will look at the novel hypothesis that important correlations between amino acid reactivities and the emergence of each of these enzymes can be drawn. We will also discuss circumstantial evidence from modern enzymes that support this hypothesis.

A word about nomenclature: Throughout this article, we will use common abbreviations for each aaRS, using the standard three-letter codes for each amino acid followed by RS to indicate tRNA synthetase. For example, alanyl-tRNA synthetase will be abbreviated as AlaRS, and so on. GluRS and AspRS can be found in two forms, discriminating (D) and non-discriminating (ND), based on their tRNA substrate specificities. In these cases, they will be referred to as D-GluRS, D-AspRS, ND-GluRS, and ND-AspRS. ND-GluRS glutamylates both tRNA^Glu^ and tRNA^Gln^ with glutamate to produce Glu-tRNA^Glu^ and Glu-tRNA^Gln^ [12]. Similarly, ND-AspRS aspartylates both tRNA^Asp^ and tRNA^Asn^ to generate Asp-tRNA^Asp^ and Asp-tRNA^Asn^ [13]. We will also refer to non-standard amino acids using their assigned three letter codes as follows: selenocysteine (Sec), ornithine (Orn), homocysteine (Hcy), and pyrrolysine (Pyl).

## 2. Early aaRS Evolution

**Figure 2:**
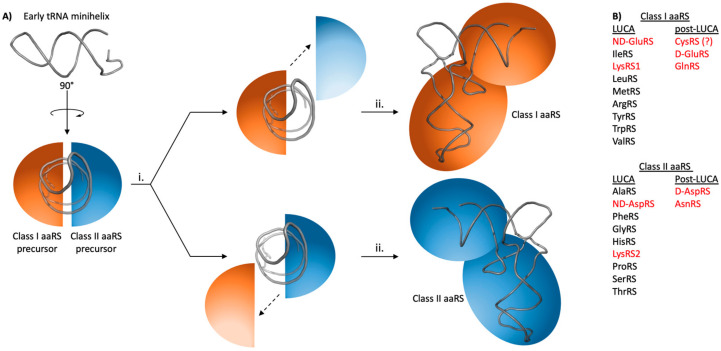
Cartoon schematic of early aaRS evolution. (**A**) A schematic summary of early aaRS evolution. Very early aaRS precursors are predicted to have aminoacylated tRNA micro- or mini- helices with substrate promiscuity. One minihelix would be recognized simultaneously by class I and class II precursors. Gene duplication (i) would have allowed these early aaRS precursors to achieve greater substrate specificity as they (ii) acquired additional domains like the anticodon-binding domains shown here to become the ancestors of modern aaRSs. Figure adapted from [8,16]. (**B**) List of aaRSs that existed at LUCA versus those that emerged post-LUCA. Enzymes discussed in this article are highlighted in red. CysRS might have existed at LUCA or emerged post-LUCA; this possibility is noted with a question mark.

Extensive work has gone into predicting, modeling, and understanding evolutionary events in the emergence and fixation of the aaRSs (Reviewed in [8]). Briefly, the two aaRS classes are believed to have evolved in parallel (Figure 2) [14,15,16]; consistently, there are ten aaRSs in each class, with the unusual exception of LysRS, which is found in both classes [9]. Each class of aaRSs was derived from a single, class-specific, active site domain. Over time, these two domains duplicated and diverged into the different precursors of modern aaRS, capable of activating amino acids and transferring the amino acid to RNA microhelices [17]. These single domains eventually acquired additional domains to achieve recognition of full-length tRNAs, for further diversification, and to acquire other functions [18]. 

Early aaRSs would have been non-specific for both their amino acid and tRNA substrates. Most proteinogenic amino acids can be synthesized under prebiotic conditions [19,20,21]. For a specific aaRS to emerge, a stable source of its cognate substrate needed to be available. For this reason, aaRS evolution had to have been closely tied to the evolution of biosynthetic pathways for the different amino acids. This concept has been considered by others in depth [22,23,24,25,26]. It will not be discussed further here except when necessary to note the probable availability of competing non-cognate substrates with respect to specific aaRSs. 

One challenge faced by many aaRSs was (and is) how to select a specific, cognate amino acid when challenged with very similar, non-cognate potential substrates. For example, an early IleRS likely aminoacylated tRNAs with both Ile and Val, and perhaps even Leu, Ala, and α-aminobutyric acid. In fact, many modern aaRSs, including IleRS, still cannot sufficiently discriminate between cognate and structurally similar non-cognate amino acids and consequently use a proofreading activity to remove non-cognate amino acids from their tRNAs [27]. These editing active sites were typically recruited to the aaRS as an appended or insertion domain; examples exist of free-standing editing enzymes as well [28]. Editing is discussed in more detail in the next section of this manuscript.

Amino acid and tRNA substrate selectivities would have become more specific over time as each enzyme evolved. By the time of LUCA, 16 of the 18 aaRSs had presumably achieved sufficient levels of substrate selectivity to allow for accurate translation of the genetic code [8,29]. ND-AspRS and ND-GluRS were the two exceptions: These two enzymes had achieved amino acid selectivity for Asp and Glu, respectively, even as they each aminoacylated tRNAs from two different isoacceptor sets (tRNA^Asp/Asn^ and tRNA^Glu/Gln^, respectively) [11].

**Figure 3:**
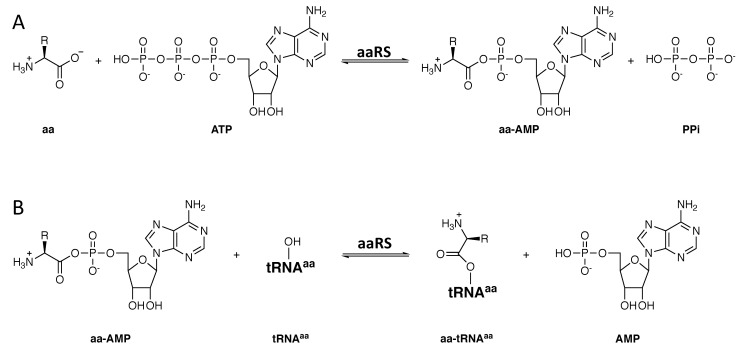
Reactions catalyzed by all known aaRSs. (**A**) Amino acid activation. (**B**) tRNA aminoacylation. Aminoacylation occurs on either the 2′ or the 3′ hydroxyl group of the 3′ ribose on the tRNA. For clarity, only a single hydroxyl group is shown.

Modern aaRSs all catalyze the same two reactions to attach their cognate amino acids to their cognate tRNA(s) (Figure 3) [30]. In step 1, the cognate amino acid is activated via condensation with ATP and the loss of inorganic pyrophosphate (PPi). Next, the activated amino acid is transferred to the 3′ ribose on the cognate tRNA. Class I aaRSs typically aminoacylate the 2′ hydroxyl on this ribose, whereas class II enzymes favor the 3′ hydroxyl group [31]. In vivo, amino acid activation is presumably driven forward by the action of inorganic pyrophosphatase, which hydrolyzes PPi into two phosphate ions [32]. The second reaction, tRNA aminoacylation, is considered irreversible at least partly because elongation factor TU (EF-Tu) binds the aminoacyl-tRNA (aa-tRNA) product. Without EF-Tu, many aa-tRNAs are unstable, as they are sensitive to hydrolysis [33]. These reactions were fixed in both aaRS classes before LUCA.

By necessity, the aminoacyl-adenylate (aa-AMP) intermediate common to all tRNA aminoacylation reactions is highly reactive. The high electrophilicity of the amino acid carbonyl, coupled with AMP as a powerful leaving group, are essential to enable nucleophilic attack by the appropriate tRNA hydroxyl group. However, we will argue herein that this reactivity prevented ornithine and homocysteine from entering the genetic code and contributed to the delay of selenocysteine’s inclusion in the modern genetic code. Amino acid side chain reactivity will also be considered as an explanation for the late emergence of AsnRS and GlnRS post-LUCA.

## 3. Generalities on How the aaRSs Recognize Cognate and Reject Non-Cognate Amino Acids

Protein translation occurs with errors at specific codons with frequencies that range from approximately 1 in 3000 to as much as 1 in 28,000 for a specific codon [34,35]. The aaRSs are prime drivers of this accuracy in that they are the enzymes that correctly pair each amino acid to its corresponding tRNA. Consequently, each aaRS had to evolve an active site that was optimized for recognition of its cognate amino acid over structurally similar non-cognate metabolites. This discrimination is straightforward in some cases and difficult in others.

Consider IleRS: its active site is tailored to select isoleucine, and thus, it would be too hydrophobic and small to allow binding of an amino acid such as arginine, for example. On the other hand, this same active site would have a much harder time discriminating against the smaller, structurally similar valine. In fact, Linus Pauling argued that binding energies would differ by only ~1 kcal/mol when the difference in size between two potential substrates was only one methylene group [36]. Consistently, IleRS misactivates valine about once for every 180 times it correctly activates isoleucine and it also misacylates tRNA^Ile^ with valine. To address this challenge, IleRS contains a second, editing active site that corrects for these errors such that valine is misincorporated into proteins at isoleucine codons with a frequency of only 1 in 3000 [37,38].

This editing strategy was first described as a double-sieve by Alan Fersht: the first sieve rejects highly dissimilar amino acids but, by necessity, lets through structurally similar ones; then, the second sieve rejects the cognate aminoacyl-tRNA while binding to and hydrolyzing any non-cognate aminoacyl-tRNAs [39]. With IleRS, the first sieve is the aminoacylation active site that occasionally misactivates valine and produces Val-tRNA^Ile^. The second sieve is the editing active site that hydrolyzes Val-tRNA^Ile^ but leaves the larger Ile-tRNA^Ile^, the desired product, intact [37,38]. This strategy is used by a number of aaRSs to ensure accurate tRNA aminoacylation [40]. Free-standing editing enzymes have also been discovered [41]. Some aaRS active sites can also release misactivated aa-AMPs, which are subsequently hydrolyzed spontaneously or via intramolecular cyclization, a process that will be discussed further below [42].

This IleRS example highlights the challenges faced by aaRSs early in evolution. Emergence of a single active site that rejects structurally dissimilar amino acids would not always have been straightforward. Before editing domains were available, evolving enzymes would have been limited to discrimination by induced fit and steric hindrance, coupled with matched polarities between the active site and the desired substrate. To our knowledge, the role of chemical reactivity in different amino acids has not been considered with respect to how the genetic code was selected.

## 4. The Evolution of LysRS: Why Is Ornithine Not Part of the Genetic Code?

As discussed above, presumably the aaRSs showed substrate promiscuity early in their evolution, becoming more selective over time until they reached their modern levels of substrate specificity. Assuming this hypothesis is true, we are interested in exploring possible features that promoted the selection of one amino acid over another. With respect to LysRS, why was lysine chosen over ornithine? Both lysine and ornithine would have been metabolically available, the latter because it is a precursor to arginine and proline [43]. Furthermore, there are modern examples of other types of promiscuous enzymes that utilize both lysine and ornithine [44], demonstrating that this type of substrate duality is possible. Ornithine is a negligible inhibitor of both LysRS1 and LysRS2 with *K_i_* values in the low mM range. LysRS2 (but not LysRS1) can aminoacylate tRNA^Lys^ with ornithine, producing the misacylated Orn-tRNA^Lys^, at least with mM concentrations of ornithine [45]. The ability of modern LysRS2 to use ornithine as a substrate, albeit weakly, supports the hypothesis that both Lys and Orn were acceptable substrates for LysRS early in evolution. So, why and how was lysine selected over ornithine for inclusion in the genetic code?

A few possibilities suggest themselves as answers to this question. First, lysine and LysRS may have been paired and selected by chance, simply winning the evolutionary coin toss. While possible, this scenario is belied by the fact that both LysRS1 and LysRS2 separately developed the ability to discriminate against ornithine in favor of lysine. Second, the use of ornithine in the biosynthesis of arginine and proline might have limited ornithine availability. However, at least in modern organisms, ornithine is part of the urea cycle; is often used in the biosynthesis of antibiotics, siderophores, and polyamines; and free ornithine was recently discovered to bind to the ribosome to regulate polyamine biosynthesis [43]; all of which suggest that ornithine availability is rarely limited. Third, differences in the chemical reactivities of ornithine versus lysine might have favored lysine. Yet, the only difference between these two amino acids is in the length of the side chain, with lysine being one methylene group longer than ornithine. This small difference only allows for discrimination against ornithine at a rate of 1 in ~200, significantly worse than the 1 in 3000 errors typically quoted as necessary for cellular viability [46]. Consequently, any reasonable hypothesis would have to explain how this difference in length could lead to more robust rejection of ornithine by LysRS.

**Figure 4:**
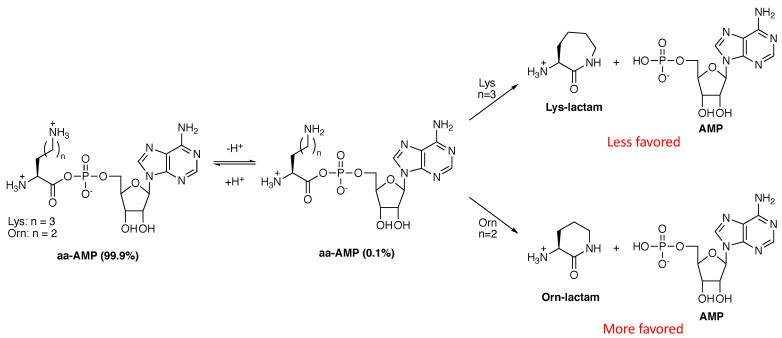
Cyclization reactions of Lys-AMP and Orn-AMP to the corresponding lactams. These intramolecular cyclization reactions would occur only when the ornithine and lysine side chains are deprotonated. Ring formation would further drive deprotonation. Formation of the six-membered ring Orn-lactam is favored ~500:1 over that of the seven-membered ring in the Lys-lactam [47,48].

We propose that the shorter length of ornithine disfavored its uptake into the code because the Orn-AMP intermediate would be intrinsically less stable than the corresponding Lys-AMP. Under physiological conditions, approximately 0.1% of the ornithine and lysine side chain amines are deprotonated, neutral, and nucleophilic at a given time. AaRS-catalyzed activation of each amino acid as an aa-AMP would position these neutral amines for intramolecular nucleophilic attack onto their own carboxylic acids, now activated for attack by AMP, to produce the corresponding lactams (Figure 4). Cyclization would pull the deprotonation equilibrium forward, further favoring cyclization. These lactam products would be highly stable due to the newly formed amide bond and their formation would likely be spontaneous.

The cyclization reactions in Figure 4 highlight an important distinction between lysine and ornithine: The lysine lactam is a seven-membered ring, and therefore disfavored by approximately 250–500-fold (~3 kcal/mol), compared to the six-membered ring formed from ornithine [47,48]. We propose that this difference was sufficient to favor ornithine lactam formation over Orn-tRNA^Lys^ biosynthesis, allowing lysine to claim a spot in the modern genetic code. In fact, modern LysRS2 has retained the ability to activate ornithine to Orn-AMP, which then cyclizes to the lactam [49]. To our knowledge, the ability of LysRS1 to make this lactam has never been studied. Given that amino acids in aminoacyl-tRNAs are activated at their carbonyl groups, lactam formation could even occur on Orn-tRNA^Lys^, further favoring the evolution of LysRS over an OrnRS. In fact, it is likely that cyclization to the ornithine lactam prohibited ornithine from consideration even earlier in evolution. In one effort to recapitulate how peptide bonds might have formed spontaneously under prebiotic conditions, lysine was readily incorporated into peptides and depsipeptides under conditions where ornithine and diaminobutyric acid (a serine isostere) cyclized to their corresponding lactams [23]. This important observation strongly suggests that amino acid side chain chemistry impacted evolution from the very beginning.

Importantly, as discussed in the next section, this same kind of side chain reactivity and cyclization can be used to explain how homocysteine might have been eliminated from the genetic code as well. The parallels between these two examples are striking, which, in our opinion, lends them further support.

## 5. The Cases against Homocysteine, Homoserine, and Selenocysteine

Differences in amino acid side chain chemistry can also be invoked to explain the exclusion of homocysteine (and possibly the late and only partial inclusion of selenocysteine) into the genetic code as well. Like ornithine, homocysteine is a metabolically available amino acid as it is a precursor to methionine and a downstream hydrolysis product from S-adenosyl methionine (SAM) methylation reactions. So, why did CysRS and MetRS specifically emerge instead of a homocysteinyl-tRNA synthetase (HcyRS) replacing one of these enzymes?

**Figure 5:**
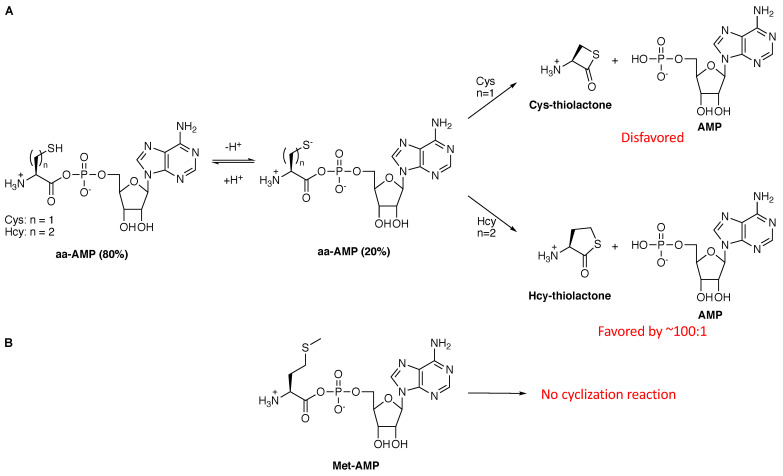
Cys-AMP and Hcy-AMP, but not Met-AMP, can degrade via cyclization. (**A**). When deprotonated, both Cys-AMP and Hcy-AMP can cyclize to their respective thiolactones. The five-membered ring formed by homocysteine is favored over the four-membered ring formed by cysteine [48]. (**B**) The methionine side chain is not nucleophilic under physiological conditions and so a similar cyclization reaction will not occur.

The side chain of homocysteine is nucleophilic, especially when deprotonated. Homocysteine’s side chain pKa is ~8, such that at physiological pH approximately 20% will be deprotonated to the highly nucleophilic sulfur anion. With the assumption that primordial CysRS and MetRS enzymes were sloppy and could activate homocysteine in addition to cysteine or methionine, respectively, then the immediate cyclization of the Hcy-AMP intermediate to the five-membered cyclic thiolactone would have been spontaneous (Figure 5A) and would have been favored over cyclization of Cys-AMP to the more strained four-membered ring by approximately 100-fold [48]. Thus, this deleterious cyclization reaction would have favored cysteine selectivity over homocysteine. Steric hindrance would have likely played an additional role in CysRS evolution. It is much easier for an enzyme active site to reject a larger amino acid, namely homocysteine, over the smaller cysteine via simple steric hindrance. Consequently, it is likely that both factors, the favorability of Hcy-AMP to cyclize and the use of steric hindrance to weaken recognition of Hcy, played roles in the emergence of CysRS over a HcyRS.

While the hydroxyl group of homoserine is significantly less nucleophilic than the thiols of cysteine and homocysteine, a similar cyclization reaction (not shown) to the homoserine lactone could also explain the absence of homoserine and a homoseryl-tRNA synthetase from the genetic code. In fact, homoserine is activated to Hse-AMP by modern LysRS2 [50], ValRS, and IleRS [51]. In all three cases, Hse-AMP is subsequently cyclized to the corresponding lactone, supporting this hypothesis. It has been proposed that these cyclization reactions are enzyme-catalyzed [52]; although, to our knowledge, mutations that disrupt this reaction without disrupting amino acid activation have never been identified, suggesting that these are examples of selective release and spontaneous cyclization instead.

MetRS would have faced a more difficult specificity challenge that is reminiscent of that faced by LysRS. Homocysteine is the metabolic precursor to methionine; it lacks the δ-methyl group and terminates in a thiol. Consequently, discrimination ability based on size would be limited, as predicted by Pauling [36] and demonstrated for IleRS [39] and other editing aaRSs (see above). However, Met-AMP would be dramatically more stable than Hcy-AMP because the sulfur in the side chain of Met is not nucleophilic in aqueous medium and thus not susceptible to degradation via cyclization (Figure 5B). (The sensitivity of methionine to oxidation will be discussed at the end of this article). Consequently, the favorability of the Hcy-AMP intramolecular cyclization reaction (Figure 5A) was the likely driving force behind MetRS acquiring specificity for methionine over homocysteine. Remarkably, support for this hypothesis is evident in modern MetRS. Modern MetRS enzymes still misactivate homocysteine to the Hcy-AMP, which cyclizes to the thiolactone [53]. It is likely that this cyclization reaction is an example of selective release and spontaneous cyclization rather than an enzyme-catalyzed editing reaction. MetRS actually releases some Met-AMP as well, suggesting that some release of Met-AMP is necessary to ensure release of Hcy-AMP to limit production Hcy-tRNA^Met^ [54]. Thus, MetRS is a modern example of the use of selective release to achieve substrate selectivity, a trait that presumably emerged pre-LUCA during the selection of methionine over homocysteine as its amino acid substrate.

The use of selenocysteine in the proteome also fits into this picture. Selenocysteine was incorporated into the genetic code as the 21st amino acid post-LUCA [55,56,57]. This cysteine isostere is ribosomally incorporated into only a few proteins in various organisms across all domains of life, including humans. In these species, the UGA stop codon has been reassigned to selenocysteine. Selenocysteine is a useful amino acid with a lower p*K*_a_ than cysteine (p*K*_a_ = 5.4); it is fully deprotonated at physiological pH [56]. It also has a lower reduction potential, making it useful for enzyme-catalyzed redox reactions, and is resistant to permanent oxidative damage [56]. Given that the more electron-rich selenide anion is a stronger nucleophile than the cysteine sulfide anion, cyclization to the selenolactone would likely have competed with tRNA aminoacylation, even with the more strained, four-membered ring as a product. Thus, it is probable that cyclization to the selenolactone contributed to the delayed use of selenocysteine in biology. In fact, in modern organisms, Sec-tRNA^Sec^ is biosynthesized indirectly after tRNA aminoacylation. This more convoluted approach to tRNA aminoacylation circumvents the formation of the highly unstable Sec-AMP to support this evolutionary scenario. Consistently, Sec-tRNA^Sec^ would also be unstable, compared to Cys-tRNA^Cys^. In fact, it is impressive that the unusual chemical capabilities of selenocysteine were sufficient to overcome the challenges of stably attaching this amino acid to tRNA^Sec^ in any organism.

## 6. The Post-LUCA Emergence of GlnRS and AsnRS

In the two preceding sections, we argued that the nucleophilicity of ornithine, homocysteine, homoserine, and selenocysteine directly impacted the exclusion of these amino acids from the standard genetic code. The emergence of GlnRS and AsnRS, post-LUCA, is harder to explain even as these two enzymes were clearly the last two to emerge and are the rarest of the 20 standard aaRSs [11]. GlnRS specifically appeared in eukarya; it is only present in a small subset of microorganisms, such as *Escherichia coli*, as a result of lateral gene transfer [58]. AsnRS emerged earlier than GlnRS and appeared in all three domains in life [59,60]. In organisms that are missing AsnRS and/or GlnRS, Asn-tRNA^Asn^ and/or Gln-tRNA^Gln^ are biosynthesized indirectly, with amino acid formation occurring on the tRNA using Asp-tRNA^Asn^ and Glu-tRNA^Gln^ as precursors (synthesized by non-discriminating versions of AspRS and GluRS) [61]. This indirect pathway was fixed at the time of LUCA [11].

The same chemistry used above to explain the exclusion of homocysteine and ornithine does not cleanly explain the very late evolution of AsnRS and GlnRS. The challenge is that the carboxylate side chains of aspartate and glutamate are much more nucleophilic than the carboxyamides of asparagine or glutamine. Thus, Asp-AMP and Glu-AMP would have been more prone to intramolecular cyclization than Asn-AMP and Gln-AMP. Any argument based on amino acid nucleophilicity would suggest that AspRS and GluRS should have emerged after AsnRS and GlnRS, instead of the other way around. We will discuss a few other possibilities here although none prove to be as satisfying as those presented above for non-coded amino acids with nucleophilic side chains.

**Figure 6:**
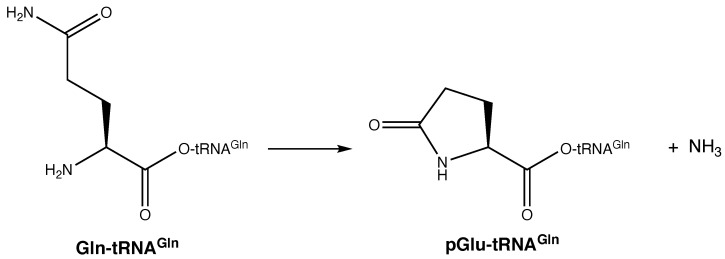
Spontaneous cyclization of glutamine to pyroglutamate. This reaction can occur spontaneously in solution (not shown) or following aminoacylation of tRNA^Gln^ (shown).

Glutamine is commonly used as a source of nitrogen throughout metabolism and was available early in evolution [62]. Consequently, a lack of glutamine cannot be used to explain the delayed evolution of GlnRS. Glutamine has another unusual property: It is prone to spontaneous cyclization to pyroglutamate (pGlu, Figure 6) [63]. In multicellular and some single cellular eukaryotes, pyroglutamate can be found on the N-terminus of some peptides and proteins. It can form spontaneously or be enzymatically synthesized by the enzyme glutaminyl cyclase [64]. It can be removed via the action of pyroglutamyl peptidase [65]. If glutamine cyclization were to inadvertently occur on tRNA^Gln^, then the resultant pGlu-tRNA^Gln^ ester bond would be stabilized (similar to acetylation of aminoacyl-tRNAs [66]); the α-nitrogen of pyroglutamate would be tied up in an amide bond such that it could not participate in ribosomal translation; and, critically, the tRNA would be permanently taken out of the translation cycle. Thus, the formation of pGlu-tRNA^Gln^ would have been energetically costly to the emerging protein translation apparatus. Pyroglutamate peptidase has been found in archaea that lack glutaminyl cyclase, and its function in these species is unknown [67]. One unexplored possibility is that it is a pGlu-tRNA^Gln^ hydrolase, such that it would free up tRNA^Gln^ for recycling back into translation.

At face value, the formation of pyroglutamate on tRNA^Gln^ seems like it could explain the delayed emergence of GlnRS. Pyroglutamate formation has been observed in assays of the archaeal amidotransferase GatDE that converts Glu-tRNA^Gln^ into Gln-tRNA^Gln^ [68,69], highlighting that Gln cyclization can occur on tRNA^Gln^. However, there are weaknesses to this theory: Glutamate can also cyclize to pyroglutamate [63] and the indirect biosynthesis of Gln-tRNA^Gln^ proceeds through a γ-phosphorylglutamatyl-tRNA^Gln^ intermediate [70,71], which would be highly prone to cyclization to pGlu-tRNA^Gln^ with phosphate as a potent leaving group. Despite these challenges, both GluRS and the indirect pathway for Gln-tRNA^Gln^ biosynthesis were fixed pre-LUCA. Consequently, it seems unlikely that deleterious pyroglutamate formation was the main driver behind delaying the emergence of GlnRS. Nevertheless, it is possible that this cyclization reaction contributed to this delay to some extent.

The corresponding asparagine cyclization reaction would yield 4-oxoazetidine-2-carboxylic acid. To our knowledge, this four-membered ring has never been observed on tRNA^Asn^, suggesting its formation was not a strong deterrent of AsnRS emergence. Additionally, aspartic acid would be prone to this same cyclization reaction. Computational calculations suggest that cyclization of aspartic acid (and asparagine by analogy) to this azetidine is not favored [72]. Together, these observations suggest that asparagine cyclization was not a driving force in development of AsnRS. In fact, the delayed emergence of AsnRS probably has little to do with amino acid chemistry. Instead, it is more likely that indirect tRNA aminoacylation of tRNA^Asn^ provided an early route to asparagine biosynthesis. It was not until the emergence of asparagine synthetase A (AS-A), which evolved from AspRS, that selection of an AsnRS could occur. Some modern organisms still lack AS-A and rely on indirect tRNA aminoacylation for asparagine biosynthesis even in the presence of a functional AsnRS [11,60].

All told, amino acid reactivities do not seem to be the major driving forces between the late fixation of GlnRS and AsnRS and other theories provide more satisfying possible explanations. For example, in *Mycobacterium tuberculosis,* indirect tRNA aminoacylation of tRNA^Asn^ can be co-opted to introduce aspartate into proteins at asparagine codons when faced with antibiotic stress [73]. Perhaps this flexibility offered enough of a selective advantage to promote the retention of indirect tRNA aminoacylation over the emergence of AsnRS and GlnRS.

## 7. Conclusions

Herein, we have examined the possible impact of amino acid side chain chemistry on the emergence of the aaRSs pre- and post-LUCA. It seems probable that chemical reactivity prohibited homocysteine, homoserine, and ornithine from entering the genetic code and delayed and limited the use of selenocysteine to a few specialized examples. We have presented these chemical arguments with respect to fixation of the modern aaRSs. However, it is quite possible that the propensities of ornithine and homocysteine to cyclize when activated at their α-carbonyls eliminated these amino acids from an emerging genetic code much earlier in time, perhaps even during the RNA world stage of evolution. Chemical reactivity might have contributed to the delay of GlnRS post-LUCA, whereas AsnRS was likely delayed only by a need for a mechanism to synthesize asparagine. The advantages of substrate promiscuity during indirect tRNA aminoacylation might also have been a factor.

We have made these arguments using organic chemistry fundamentals paired with circumstantial evidence from phylogenetic analyses and studies on extant aaRSs and related enzymes. With all evolutionary theories for events that might have occurred pre-LUCA, it is difficult to imagine being able to prove the hypotheses proposed here. Computational calculations could provide energies of activation for the different reactions discussed. Different aa-AMPs could be synthesized and their rates of cyclization could be directly measured; these experiments are challenging, however, given the intrinsic lability of these intermediates. Finally, an archaeal pyroglutamate peptidase could be tested for pGlu-tRNA^Gln^ hydrolase activity. Experiments like these would offer further circumstantial support for these hypotheses.

We also chose to focus only on systems that show unusual phylogenetic patterns (e.g., LysRS, CysRS) or were missing in LUCA (GlnRS, AsnRS, Sec-tRNA^Sec^, maybe CysRS). Other chemical challenges could have impacted aaRS evolution pre-LUCA. Notably, oxidative damage might have posed a challenge that could have impacted cysteine, methionine, tryptophan and tyrosine availability and stability [74]. Nevertheless, the corresponding aaRSs, with the possible exception of CysRS, all evolved pre-LUCA. 

To our knowledge, amino acid reactivity has been largely neglected from evolutionary considerations of the aaRSs. This omission has neglected the important role that chemistry likely played in early selection of these enzymes. 

## Data Availability

Not applicable.
